# Exploring the impact of light intensity under speed breeding conditions on the development and growth of lentil and chickpea

**DOI:** 10.1186/s13007-024-01156-9

**Published:** 2024-02-18

**Authors:** Mohammed Mitache, Aziz Baidani, Bouchaib Bencharki, Omar Idrissi

**Affiliations:** 1 Laboratory of Food Legumes Breeding, Regional Center of Agricultural Research of Settat, National Institute of Agricultural Research, Avenue Ennasr, BP 415 Rabat Principale, 10090 Rabat, Morocco; 2 Laboratory of Agrifood and Health, Faculty of Sciences and Techniques, Hassan First University of Settat, BP 577, 26000 Settat, Morocco

**Keywords:** Food legumes, Speed breeding, Extended photoperiod, Light intensity, Lentil, Chickpea

## Abstract

The use of high-performant varieties could help to improve the production of food legumes and thus meet the demand of the growing world population. However, long periods needed to develop new varieties through traditional breeding are a major obstacle. Thus, new techniques allowing faster genetic advance are needed. Speed breeding using longer periods of light exposure on plants, appears to be a good solution for accelerating plant life cycles and generation turnover. However, applying extended photoperiod causes plant stress and mortality due to lack of information on the adequate intensity to be used in speed breeding protocol. This study examines the impact of light intensity under speed breeding conditions on the development and growth of lentils and chickpeas, with a keen interest in enhancing genetic gain in these key food legumes. Four distinct levels of light intensity (T1: Green-house: 2000 µmol/m^2^/s; T2: 148–167 µmol/m^2^/s; T3: 111–129 µmol/m^2^/s; T4: 74–93 µmol/m^2^/s) under a photoperiod of 18 h of light and 6 h of darkness were tested in a growth chamber. Significant variation depending on light intensity was observed for plant height, total biomass, number of secondary stems, pods number, number of seeds per plant, growth rate, green canopy cover, time to flowering, time of pod set, time to maturity, vegetative stage length, reproduction stage length and seed filling stage length. Light intensity significantly influenced flowering/maturity and plant’s stress compared to normal conditions in green-house where flowering/maturity were around 67/97 days for lentil and 79/111 days for chickpea. Therefore, lentils in treatment 2 flowered and reached maturity in 30/45 days respectively, with high stress, while chickpeas in the same treatment did not flower. In contrast, treatment 4 showed interesting results, promoting optimal growth with low stress, and flowering/maturity in 27/46 days and 28/54 days, respectively for lentils and chickpeas. These results underline the crucial importance of light management in speed breeding to accelerate vegetative growth and phenology while allowing healthy growth conditions for plants to produce enough seeds for generation turnover.

## Introduction

Light quality, light intensity and photoperiod duration are key regulatory factors for healthy plant growth and development in controlled environments using artificial light sources, particularly light-emitting diode lamps (LEDs). Optimum light quality, in particular the ratio of red and far-red (R:FR) to blue lights, has been extensively studied for many plant species, including radishes [1], grapes [2], lettuce [3] and lentil [4] to establish lighting conditions conducive to plant growth.

Light intensity, refers to the rate at which light spreads over a given surface area. It is also referred to as the energy transferred per unit area [5, 6]. Furthermore, the intensity of light plays a fundamental and indispensable role in the growth and development of plants. As a primary source of energy for photosynthesis [4], light provides the necessary foundation for the production of organic molecules and the development and growth of plant tissues. The importance of light intensity lies in its direct influence on the speed and efficiency of photosynthesis, the key process by which plants convert light into chemical energy [6]. Adequate light intensity ensures a sufficient supply of energy to fuel the biochemical reactions of photosynthesis, promoting the production of sugars, starch, and other metabolic compounds necessary for plant growth and development [5]. Furthermore, light intensity influences plant morphology by regulating leaf size, stem branching, root formation, and other aspects of plant architecture. Optimal light intensity allows for a balanced allocation of resources and fosters the harmonious growth of all parts of the plant. In addition to photoperiod duration, the light intensity can also influence flowering time, which decreases with increasing light intensity [7, 8]. However, excessive light intensity can also be detrimental. High levels of light can cause energy overload, leading to oxidative damage and deterioration of cellular components, and, when the intensity of light continues to rise, chlorophyll becomes vulnerable to damage, leading to a subsequent decrease in the rate of photosynthesis [6].

Plants have developed photoprotection mechanisms to protect against the damaging effects of light excess, notably in Photosystem II (PSII) [9]. When light is abundant, PSII can become overloaded, leading to oxidative damage to the vital components of this photosynthetic system. To avoid this, plants implement various photoprotection strategies to minimize damage caused by light excess [10]. One of these protective mechanisms is non-photochemical energy dissipation [9, 11]. Plants can convert the excess energy into heat rather than potentially damaging chemical reactions. Moreover, plants can also regulate the number and concentration of photosynthetic pigments present in their cells, increasing or decreasing their concentration depending on the light intensity. This adaptation enables more efficient use of light and protection against oxidative damage and it is demonstrated that metabolic responses to severe water stress and intense light occur indirectly as a result of oxidative stress, rather than being a direct response to water scarcity [12]. Additionally, some plants may exhibit shading mechanisms, where leaves or plant structures overlap to reduce direct exposure to intense light, but excessive shading can induce leaf senescence in plants exposed to low light intensities [13]. These plant adaptation mechanisms to varying light intensity demonstrate their dynamic responsiveness to environmental conditions.

Light intensity has a significant influence on metabolism and plant morphology, including leaf size, stem growth, plant height, and root development [14, 15]. When subjected to high light intensity, plants tend to develop smaller and thicker leaves. Therefore, using an optimum level of light intensity, quality and duration, and maintaining a low level of plant stress, can be beneficial. This can contribute to better plant health and reduced mortality of plants under speed breeding conditions, promoting greater stability and productivity across generations [16, 17].

To our knowledge, there are no published studies on the impact of photoperiod and light intensity during the vegetative and reproductive stages of lentil and chickpea on their growth and development. The main objective of our study is to thoroughly analyze the impact of light intensity on plant morpho-physiology and phenology. We aim to understand how different light intensities influence key processes in plants growth and development. Furthermore, we seek to assess the consequences of these variations in light intensity on plant morphology and photosynthesis. By gaining a better understanding of how light intensity affects plant morpho-physiology and phenology, we can provide valuable insights to optimize growing conditions and enhance growth, especially for food legumes such as chickpeas and lentils. Overall, our study aims to contribute to advancing knowledge in this field and provide a strong scientific foundation for practical applications aimed at improving crop production and food security.

## Material and methods

### Plant material, photo-thermal regime and experimental design

**Fig. 1:**
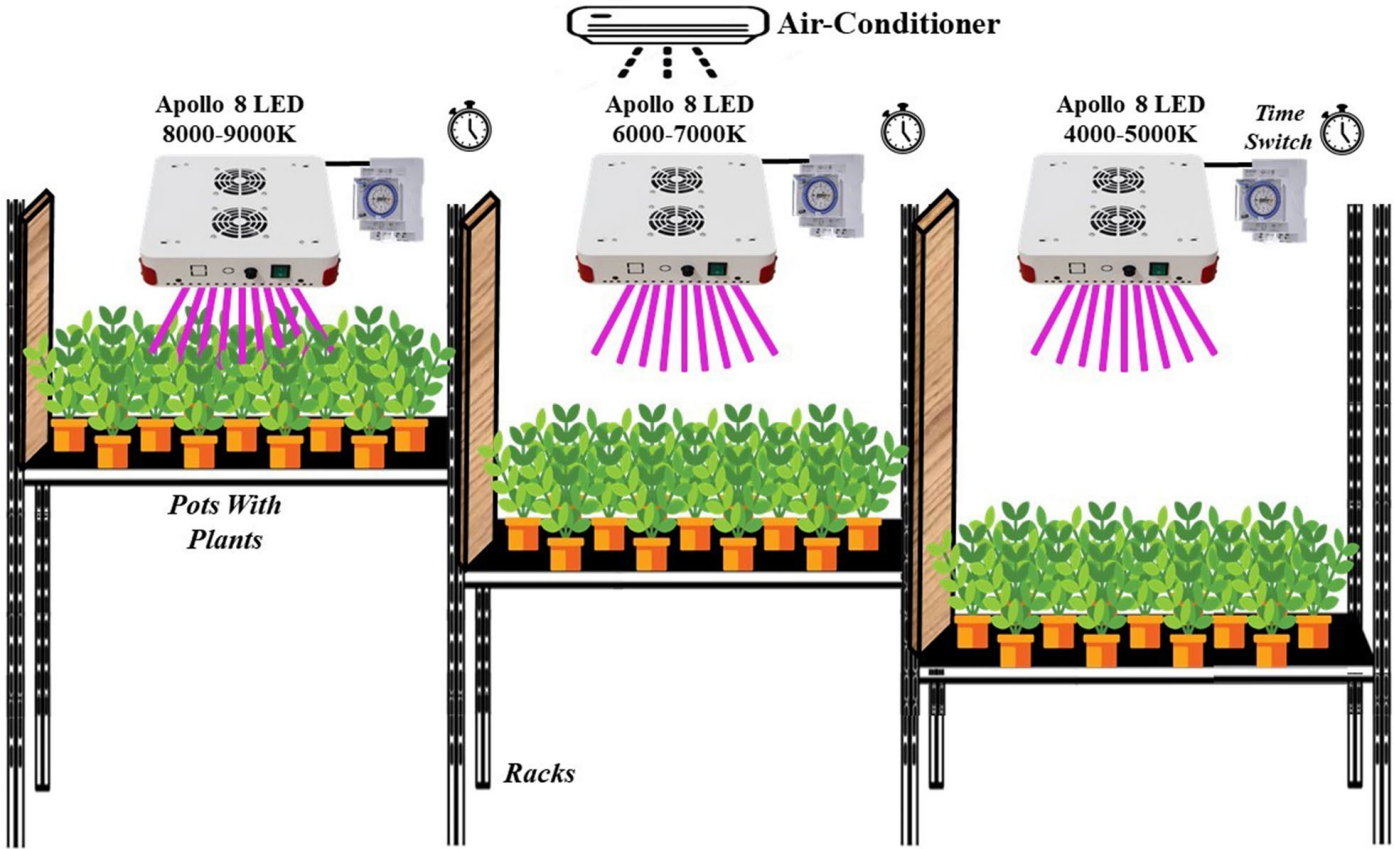
Speed breeding growth chamber design with different light intensities

Two genotypes of both lentil (*Lens culinaris* M.) [Bakria and L24] and chickpea (*Cicer arietinum*) [Farihane and Douyet] were selected from the germplasm of the genetic improvement programs of the National Institute for Agricultural Research (INRA Morocco). The selected genotypes were grown under controlled conditions with a photoperiod treatment consisting of 18 h of light at temperatures ranging from 23 to 25 °C and 6 h of darkness at temperatures ranging from 14 to 16 °C in a speed breeding growth chamber where the light source was ‘APOLLO 8’ broadband lamps (410–730 nm) with an output of 240 W. The control treatment was carried out under green-house conditions with 10–14 h of the daylight between January to June. The experiment involved the implementation of four different light intensities: 2000 µmol/m^2^/s under greenhouse conditions (treatment 1), 148–167 µmol/m^2^/s (treatment 2, speed breeding growth chamber), 111–129 µmol/m^2^/s (treatment 3, speed breeding growth chamber), and 74–93 µmol/m^2^/s (treatment 4, speed breeding growth chamber). To effectively examine the impact of these light intensities, a split plot design was employed with light intensity serving as the main factor and genotype as the sub-plot factor with 3 replications (Fig. 1).

For the four treatments, three seeds of each variety were planted in plastic pots of 5 L capacity filled with 2/3 soil and 1/3 peat compost. The pots were watered every 4–7 days based on the growth stage of the crops and its corresponding water consumption using same quantity of water for all pots.

### Plant growth and development monitoring

The studied morphological, physiological and phenological traits and measurements taken during this study were examined in detail (Table 1).

**Table 1:** Morphological, phenological and physiological traits scored on lentil, chickpea varieties under different light intensities

Traits scored	Abbreviations	Description and time scored
Empty pods percentage	EPP	Measured by counting the number of empty pods per plant after plant harvesting
Green canopy cover	GCC	Measured using the Canopeo application/tool
Growth rate	GR	Measured by the difference in the height of the main stem at 2 and 3 after emergence
Number of secondary stems per plant	NSS	Measured by counting secondary shoots after plant harvesting
Number of seed per plant	NSP	Measured by counting the number of seed per plant after plant harvesting
Plant height	PH	Measured with a graduated ruler after plant harvesting by (cm)
Pods number	PN	Measured by counting the number of pods per plant after plant harvesting
Reproduction stage length	RPS	Measured by determining the interval between the day of the first flower's appearance and that of the first pod's appearance
Seed filing stage length	SFS	Measured by determining the interval between the day of the first pods and that of its physiological maturity
Seedling vigor 1	SV1	Measured at vegetative stage using scoring system described by [18]
Plant vigor 2	SV2	Measured at flowering stage using scoring system described by [18]
Plant vigor 3	SV3	Measured at pod setting stage using scoring system described by [18]
Plant vigor 4	SV4	Measured at maturity stage using scoring system described by [18]
Time to pod set	TPS	Measured by counting the days from emergence to appearance of the first pod
Time to flowering	TF	Measured by counting the days of plant emergence to the day of the first flower's appearance
Time to maturity	TM	Measured by counting the days from emergence to the yellowing and desiccation of the plant and the pods
Total biomass	TB	Measured weighing all aerial plant parts with a balance after plant harvesting
Vegetative stage length	VGS	Measuring by determining the interval between the day of emergence and the day of appearance of the first flower
Wilting severity 1	WS1	Measured at vegetative stage using the scoring scale described by Singh et al., 2013
Wilting severity 2	WS2	Measured at flowering stage using the scoring scale described by Singh et al., 2013
Wilting severity 3	WS3	Measured at pod setting stage using the scoring scale described by Singh et al., 2013
Wilting severity 4	WS4	Measured at maturity stage using the scoring scale described by Singh et al., 2013

### Statistical analysis

Descriptive statistics and two-way analysis of variance, with light intensity and variety as factors, were performed to evaluate the impact of different light intensities, varieties and their interaction on the measured variables. Statistical Package for the Social Sciences (SPSS) version 21 was used for descriptive statistics, while R software was used for variance analysis (ANOVA) through the “agricolae” package [19]. Tukey HSD post-hoc tests were used to test the differences between the different light intensity treatments studied using the “multcomp” package [20]. In order to better understand how light intensity and variety influence the studied variables, principal component analysis was performed using the R package ‘FactoMineR, factoextra’ [21]. Graphical extrapolation of the cinetics results was performed using Microsoft Excel version 2013.

## Results

### Morphological, phenological and physiological variation in lentil varieties depending on light intensity

Highly significant variation depending on photoperiod intensity (Treatment) was observed for all studied morphological, phenological and physiological traits except empty pods percentage and wilting severity at vegetative stage (Table 2). The two lentil varieties were similar for all traits except plant height, number of seeds per plant, vegetative stage length, time to flowering, time to pod set and pods’ number, which revealed significant differences. The interaction of the two factors was significant for plant height, pods number, number of seeds per plant, growth rate, showing that the influence of light intensity depends on genotype.

**Table 2:** Analysis of variance of 22 morpho-physiological, phenological and growth traits measured for two lentil varieties (Bakria and L24), under four light intensity treatments

Source of variation	Df	NSP	GR	GCC	TF	TPS	TM	VGS	RPS	SFS	PH	TB
Treatment	3	721.5***	0.15976***	0.4774	1263.1***	1355.8***	2126.8***	1263.1***	4.593*	94.76***	107.07*	17.777***
Variety	1	247.0**	0.01722 ns	0.0360 ns	30.1*	18.8*	14.1 ns	30.1*	1.333 ns	0.33 ns	96.00	0.078 ns
Treatment:Variety	3	137.4*	0.06547*	0.1609 ns	6.7 ns	2.1 ns	4.1 ns	6.7 ns	1.333 ns	0.33 ns	64.91	0.295 ns

Source of variation	Df	NSS	PN	EPP	SV1	SV2	SV3	SV4	WS1	WS2	WS3	WS4
Treatment	3	0.253 ns	464.6***	422.9 ns	2.9306**	2.7593***	1.2037**	1.4815***	0.26389 ns	0.9444**	1.2222*	1.7222**
Variety	1	1.500 ns	273.4***	278.1 ns	0.0417 ns	0.0000 ns	0.3333 ns	0.3333 ns	0.04167 ns	0.3333 ns	0.7500 ns	0.7500 ns
Treatment: Variety	3	6.549 ns	85.9*	11.2 ns	0.3750 ns	0.0000 ns	0.3333 ns	0.3333 ns	0.15278 ns	0.0000 ns	0.0833 ns	0.7500 ns

Signif. codes: 0 ‘***’ 0.001 ‘**’ 0.01 ‘*’ 0.05 ‘.’ 0.1 ‘ns’ 1

*PH* plant height, *TB* total biomass, *NSS* number of secondary stem, *PN* pods number, *EPP* empty pods percentage, *NSP* number of seeds per plant, *GR* growth rate, *GCC* green canopy cover, *SV1* seedling vigor 1, *SV2* seedling vigor 2, *SV3* seedling vigor 3, *SV4* seedling vigor 4, *WS1* wilting score 1, wilting score 2, *WS3* wilting score 3, *WS4* wilting score 4, *TF* time to flowering, *TPS* time of pod set, *TM* time to maturity, *VGS* vegetative stage length, *RPS* reproduction stage length, *SFS* seed filling stage length

**Fig. 2:**
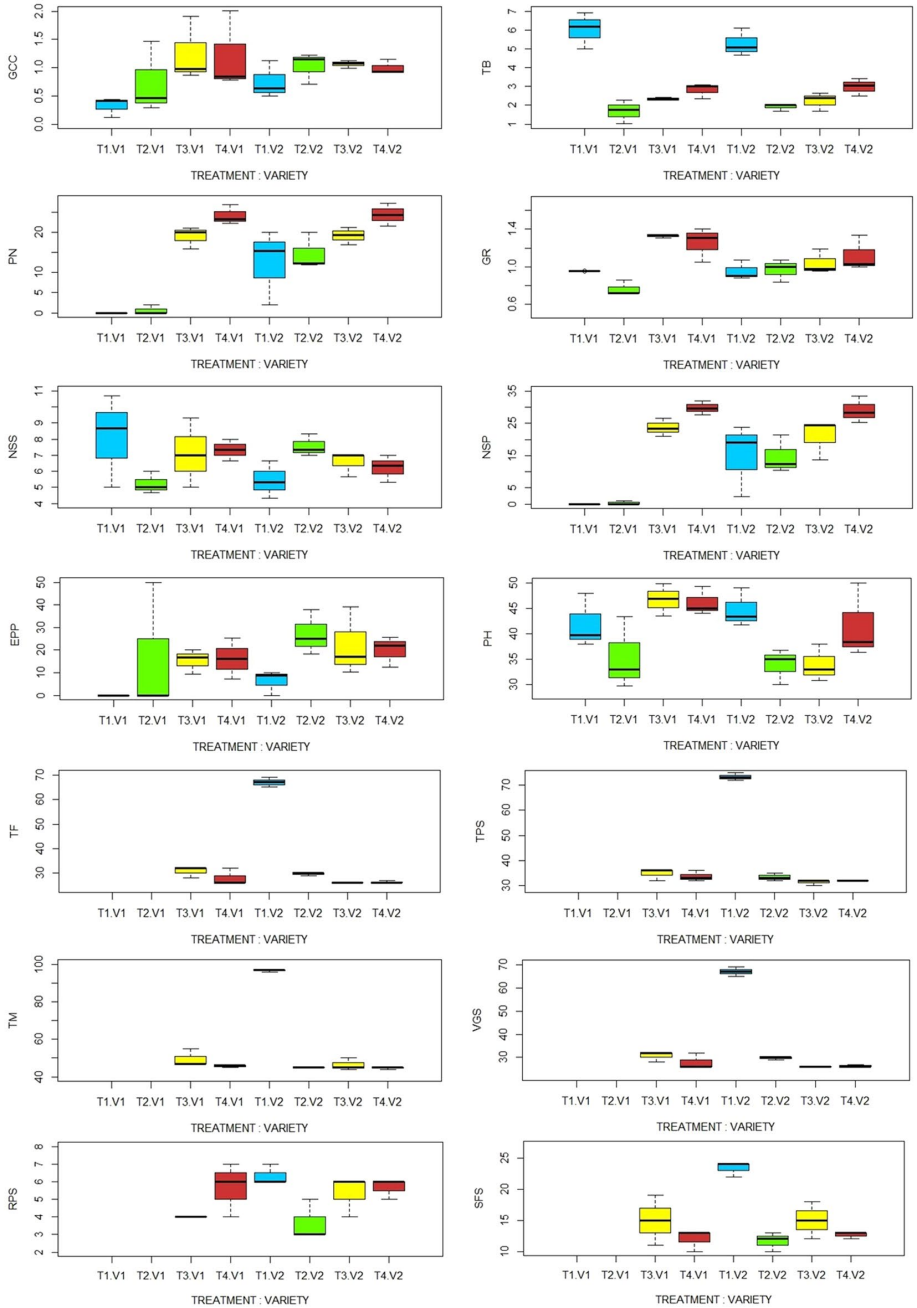
Boxplot of variance and interaction between lentil varieties and light intensity treatments. *PH* plant height, *TB* total biomass, *NSS* number of secondary stem, *PN* pods number, *EPP* empty pods percentage, *NSP* number of seeds per plant, *GR* growth rate, *GCC* green canopy cover, *TF* time to flowering, *TPS* time of pod set, *TM* time to maturity, *VGS* vegetative stage length, *RPS* reproduction stage length, *SFS* seed filling stage length

The generated boxplots show that, for each measured traits, there are significant variations between the different lentil varieties under different light intensity treatments (Fig. 2).

The influence of light intensity and spectral composition on the flowering time of the two lentil varieties (Bakria and L24) was thoroughly examined in this study. Under treatment 3 (111–129 µmol/m^2^/s) and treatment 4 (74–93 µmol/m^2^/s), variety Bakria showed the shortest flowering time, with flowering occurring 26 days after sowing. In contrast, for variety L24, the flowering time was slightly longer, taking 31 and 28 days after sowing for treatment 3 and treatment 4, respectively. It was noteworthy, to note that compared to treatments under the speed breeding growth chamber, treatment 1 (Green-house: 2000 µmol/m^2^/s) showed the longer flowering time with 67 days after sowing for Bakria variety (Table 3). Mildew fungus attacks on L24 plants under the greenhouse, caused their failure before achieving flowering. Similarly, under treatment 2 (148–167 µmol/m^2^/s), L24 plants experienced stress due to the high intensity of light, that started with burns on leaves and severe wilting followed by death preventing them from reaching the flowering stage. Additionally, treatment 2 exhibited a shorter time interval of 4 days between flowering and pod set, comparing to 6 days in treatment 4 during the reproductive stage (Table 3). These results suggest that under high light stress conditions, lentil plants tend to accelerate seed production at a faster rate.

**Table 3:** Means comparison by Tukey test for the effects of light intensity treatments on measured traits in lentil varieties

Traits											
Treatment	PH	TB	NSS	PN	EPP	NSP	GR	GCC	TF	TPS	TM
Green House	43.28a	5.65a	6.78a	6.22a	3.12a	7.5a	0.95a	0.54a	67a	73.33a	96.67a
8000–9000 Lux	34.61b	1.8b	6.39a	7.72a	21.86b	7.5a	0.87a	0.88a	29.67b	33.33b	45b
6000–7000 Lux	40.33a	2.28c	6.83a	19.11b	18.82b	22.22b	1.18b	1.16b	28.33b	33b	48b
4000–5000 Lux	43.83a	2.89c	6.78a	24.33c	18.07b	29.39b	1.19b	1.1a	27.17b	32.83b	45.57b

Traits											
Treatment	VGS	RPS	SFS	SV1	WS1	SV2	WS2	SV3	WS3	SV4	WS4
Green House	67a	6.33a	23.33a	4a	0a	4a	0a	4a	1a	4a	1a
8000–9000 Lux	29.67b	3.67b	11.67b	3b	2b	3b	1b	2b	2b	2b	3b
6000–7000 Lux	28.33b	4.67a	15b	3b	1a	4a	1b	4a	2b	4a	2a
4000–5000 Lux	27.17b	5.67a	12.33b	4a	0a	4a	0a	4a	1a	4a	1a

Letters (a, b, c, d) denote significant differences among light intensity treatments (pb0.05, Tukey’s post hoc test). nd not determined

*PH* plant height, *TB* total biomass, *NSS* number of secondary stem, *PN* pods number, *EPP* empty pods percentage, *NSP* number of seeds per plant, *GR* growth rate, *GCC* green canopy cover, *SV1* seedling vigor 1, *SV2* seedling vigor 2, *SV3* seedling vigor 3, *SV4* seedling vigor 4, *WS1* wilting score 1, wilting score 2, *WS3* wilting score 3, *WS4* wilting score 4, *TF* time to flowering, *TPS* time of pod set, *TM* time to maturity, *VGS* vegetative stage length, *RPS* reproduction stage length, *SFS* seed filling stage length

For the physiological traits of lentil varieties (Bakria and L24), including growth rate, total biomass, green canopy cover, and plant height, treatment 4 demonstrated the highest values for these traits, while treatment 2 exhibited lower values. When it came to yield-related traits, treatment 4 showed the highest number of seeds per plant and pods number, along with a lower percentage of empty pods (Table 3). Conversely, treatment 2 displayed lower values for these yield-related traits. Additionally, under treatment 2, higher plant stress was observed, as evidenced by elevated wilting scores for both Bakria and L24 varieties. On the other hand, treatment 1 resulted in lower wilting scores, indicating lower stress levels for the plants. Overall, the findings highlight the significant impact of light intensity and spectral composition on the phenological and physiological traits of lentil varieties, underscoring the importance of carefully managing light conditions to optimize flowering and yield outcomes.

### Multifactor and multivariable analysis on lentil varieties under different light intensities

**Fig. 3:**
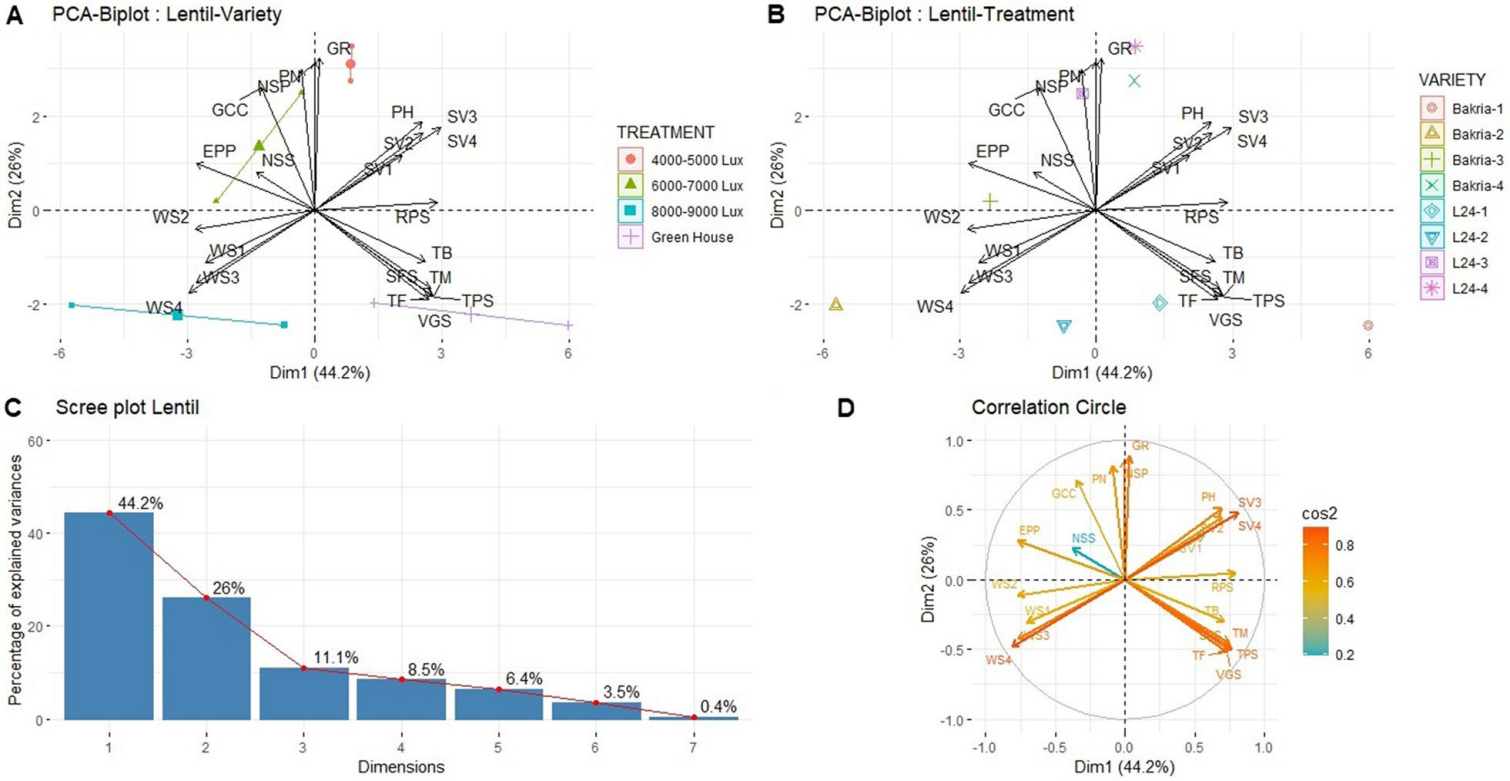
**A**–**D** Multivariable analysis on lentil varieties under light intensity treatments. *PH* plant height, *TB* total biomass, *NSS* number of secondary stem, *PN* pods number, *EPP* empty pods percentage, *NSP* number of seeds per plant, *GR* growth rate, GCC green canopy cover, *SV1* seedling vigor 1, *SV2* seedling vigor 2, *SV3* seedling vigor 3, *SV4* seedling vigor 4, *WS1* wilting score 1, wilting score 2, *WS3* wilting score 3, *WS4* wilting score 4, *TF* time to flowering, *TPS* time of pod set, *TM* time to maturity, *VGS* vegetative stage length, *RPS* reproduction stage length, *SFS* seed filling stage length

A Principal Component Analysis (PCA) was conducted to analyze all variables for each light intensity treatment (T1: Green-house: 2000 µmol/m^2^/s; T2: 148–167 µmol/m2/s; T3: 111–129 µmol/m^2^/s; T4: 74–93 µmol/m^2^/s) in relation to the two lentil varieties (Bakria and L24), considering all studied traits (Fig. 3). The PCA analysis revealed that PCA1 and PCA2 explained 44 and 26% of the total variation, respectively (Fig. 3c). The high cos2 values indicated good representation of variables near the circumference of the correlation circle, while low cos2 values suggested variables were not well represented by the main axes and were positioned closer to the center of the circle (Fig. 3d). Interestingly, the PCA plots demonstrated distinct correlation patterns for the studied traits depending on the light intensity and variety (Fig. 3b). Treatment 1 showed positive correlations with vegetative stage length, time of pods set, time to flowering, time to maturity, and seed filling stage length, indicating a positive association with phenological stages but a negative association with physiological traits and stress-related traits. Conversely, treatment 2 exhibited positive correlations with wilting scores 1, 2, 3, and 4, indicating a positive association with stress-related traits. On the other hand, treatments 3 and 4 displayed positive correlations with number of secondary stems per plant, green canopy cover, pods number, number of seeds per plant, and growth rate, suggesting a positive relationship with plant growth and physiological variables. In summary, the PCA analysis demonstrated that different light intensities had distinct effects on the correlations between phenological and physiological traits of lentil varieties. Treatments 1, 2, 3, and 4 showed specific associations with different trait categories, highlighting the importance of managing light conditions effectively to optimize both phenological and physiological.

### Morphological, phenological and physiological variation in chickpea varieties depending on light intensity

Highly significant variation depending on photoperiod intensity (Treatment) was observed for all studied morphological, phenological and physiological traits except empty pods percentage and seed filling stage length, for which insignificant effect was observed (Table 4). The two chickpea varieties were similar for all traits except empty pods percentage and seed filling stage length, which revealed significant differences. The interaction of the two factors was significant for growth rate and seed filling stage length, showing that the influence of light intensity depends on genotype.

**Table 4:** Analysis of variance of 22 morpho-physiological, phenological and growth traits measured for two chickpea varieties (Farihane and Douyet), under four light intensity treatments

Source of variation	Df	TB	GCC	GR	PH	PN	NSS	EPP	TF	TPS	TM	VGS
Treatment	3	69.79 ***	11.440 **	0.7229 ***	458.5 ***	65.19 **	5.461 *	290.0 ns	5343 ***	6594 ***	6595 ***	4054 ***
Variety	1	0.62 ns	0.002 ns	0.0004 ns	12.0 ns	9.80 ns	0.560 ns	2974.0	27 ns	18 ns	1 ns	214 ns
Treatment: variety	3	3.39 ns	0.391 ns	0.0687 **	18.5 ns	10.38 ns	0.696 ns	744.5 ns	8 ns	22 ns	0 ns	61 ns

Source of variation	Df	NSP	RPS	SFS	SV1	SV2	SV3	SV4	WS1	WS2	WS3	WS4
Treatment	3	28.684 ***	70.22 **	9.389 ns	5.486 ***	1.7222 ***	1.7222 ***	4.222 ***	4.278 ***	2.3889 **	4.667 ***	10.500 ***
Variety	1	0.116 ns	0.89 ns	29.389	0.042 ns	0.2222 ns	0.0000 ns	0.222 ns	0.000 ns	0.0000 ns	0.056 ns	0.222 ns
Treatment: variety	3	0.424 ns	5.56 ns	26.722	0.153 ns	0.0556 ns	0.1667 ns	0.222 ns	0.111 ns	0.1667 ns	0.222 ns	0.056 ns

Signif. codes: 0 ‘***’ 0.001 ‘**’ 0.01 ‘*’ 0.05 ‘.’ 0.1 ‘ns’ 1

*PH* plant height, *TB* total biomass, *NSS* number of secondary stem, *PN* pods number, *EPP* empty pods percentage, *NSP* number of seeds per plant, *GR* growth rate, *GCC* green canopy cover, *SV1* seedling vigor 1, *SV2* seedling vigor 2, *SV3* seedling vigor 3, *SV4* seedling vigor 4, *WS1* wilting score 1, wilting score 2, *WS3* wilting score 3, *WS4* wilting score 4, *TF* time to flowering, *TPS* time of pod set, *TM* time to maturity, *VGS* vegetative stage length, *RPS* reproduction stage length, *SFS* seed filling stage length

**Fig. 4:**
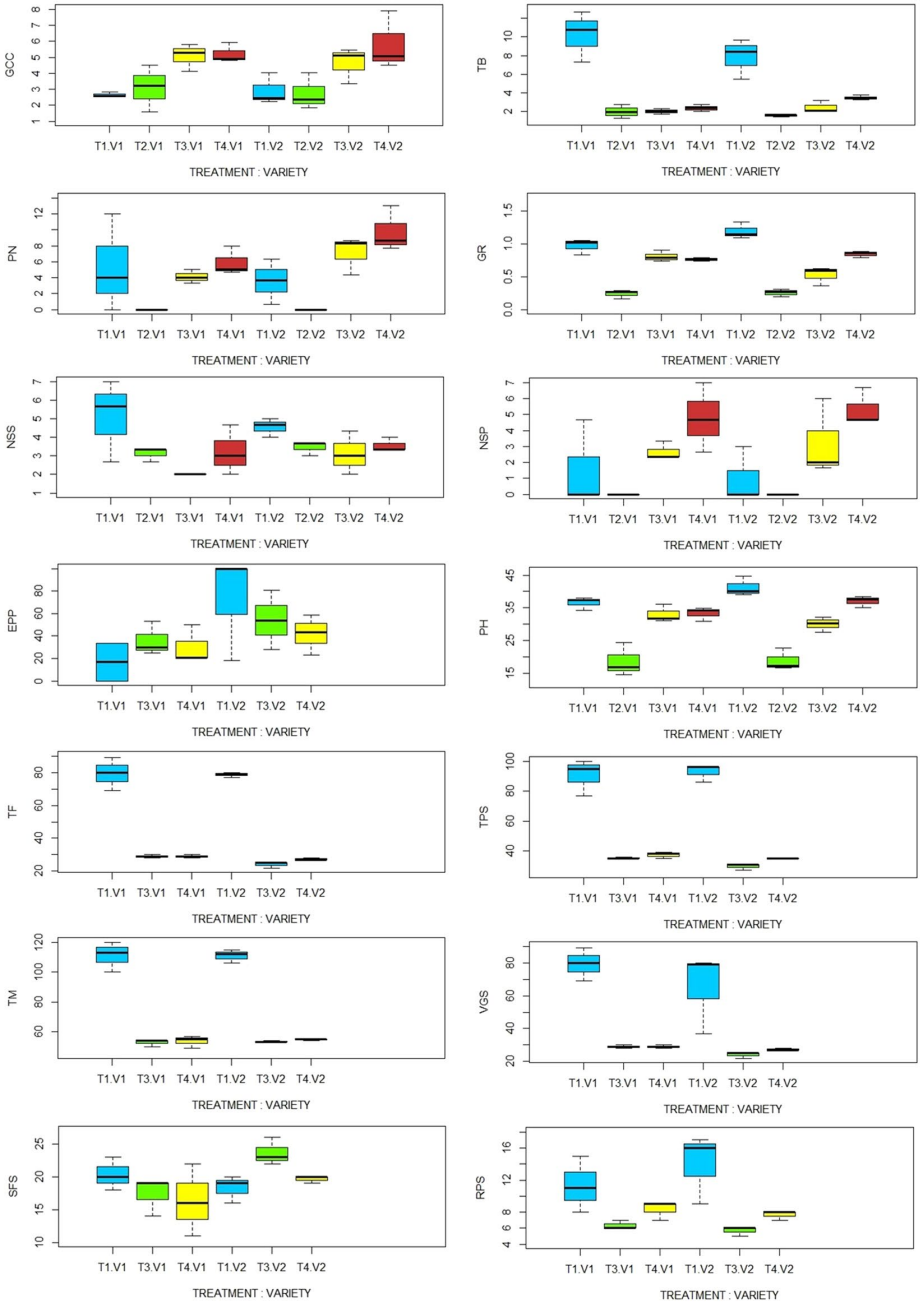
Boxplot of variance and interaction between chickpea varieties and light intensity treatments. *PH* plant height, *TB* total biomass, *NSS* number of secondary stem, *PN* pods number, *EPP* empty pods percentage, *NSP* number of seeds per plant, *GR* growth rate, *GCC* green canopy cover, *TF* time to flowering, *TPS* time of pod set, TM time to maturity, *VGS* vegetative stage length, *RPS* reproduction stage length, *SFS* seed filling stage length

The generated boxplots show that, for each measured traits, there are significant variations between the different chickpea varieties under different light intensity treatments (Fig. 4).

The influence of light intensity and spectral composition on the flowering time of chickpea varieties (Farihane and Douyet) was thoroughly examined in this study. Under treatment 3 (111–129 µmol/m^2^/s) and treatment 4 (74–93 µmol/m^2^/s), variety Farihane showed the shortest flowering time, with flowering occurring 29 and 28 days after sowing for treatment 3 and 4, respectively. In contrast, for variety Douyet, the flowering time was slightly shorter, taking 24 and 27 days after sowing for treatment 3 and treatment 4, respectively (Table 5). It was noteworthy that treatment 1 (Green-house: 2000 µmol/m^2^/s) showed the longer flowering time with 79 days after sowing for Farihane and Douyet varieties. Under treatment 2 (148–167 µmol/m^2^/s), Farihane and Douyet plants experienced stress due to the high intensity of light, preventing them from reaching the flowering stage. The same trends were observed for the pods set and maturity times. Additionally, treatment 3 exhibited a shorter time interval of 6 days between flowering and pod set during the reproductive stage, while a longer time interval of 24 days between pod set and maturity for the seed filling stage length (Table 5). These results suggest that under high light stress conditions, chickpea plants tend to accelerate seed production and achieve full maturity more slowly.

**Table 5:** Means comparison by Tukey test for the effects of light intensity treatments on measured traits in chickpea varieties

Traits											
Treatment	PH	TB	NSS	PN	EPP	NSP	GR	GCC	TF	TPS	TM
Green House	38.89a	9.06a	4.83a	4.44a	50.3a	1.28a	1.08a	2.77a	79a	91.67a	111a
8000–9000 Lux	18.78b	1.77b	3.28b	0.00b	nd	0.00a	0.25b	2.92a	nd	nd	nd
6000–7000 Lux	31.56c	2.21b	2.56b	5.61a	45.16a	2.94a	0.67c	4.85b	26.5b	32.5b	53b
4000–5000 Lux	35.17c	2.92b	3.39a	7.83a	36.06a	5.06b	5.52d	0.8b	28.17b	36.17b	54.17b

Traits											
Treatment	VGS	RPS	SFS	SV1	WS1	SV2	WS2	SV3	WS3	SV4	WS4
Green House	79a	12.67a	19.33a	5a	0a	5a	0a	5a	1a	5a	1a
8000–9000 Lux	Nd	nd	nd	3b	2b	nd	nd	nd	nd	nd	nd
6000–7000 Lux	26.5b	6b	20.5b	4c	2b	4b	1b	4b	2b	3b	3b
4000–5000 Lux	28.17b	8b	18c	4c	2b	4b	1b	4b	2b	4c	3b

Letters (a, b, c, d) denote significant differences among light intensity treatments (pb0.05, Tukey’s post hoc test), nd not determined

*PH* plant height, *TB* total biomass, *NSS* number of secondary stem, *PN* pods number, EPP empty pods percentage, *NSP* number of seeds per plant, *GR* growth rate, *GCC* green canopy cover, *SV1* seedling vigor 1, *SV2* seedling vigor 2, *SV3* seedling vigor 3, *SV4* seedling vigor 4, *WS1* wilting score 1, wilting score 2, *WS3* wilting score 3, *WS4* wilting score 4, *TF* time to flowering, *TPS* time of pod set, TM time to maturity, *VGS* vegetative stage length, *RPS* reproduction stage length, *SFS* seed filling stage length

For the physiological traits of chickpea varieties (Farihane and Douyet), including growth rate, total biomass, green canopy cover, and plant height, treatment 2 demonstrated the lower values for these traits. When it came to yield-related traits, treatment 4 showed the highest number of seeds per plant and pods number, along with a lower percentage of empty pods (Table 5). Additionally, under treatment 2, higher plant stress was observed, as evidenced by elevated wilting scores for both Farihane and Douyet varieties. On the other hand, treatment 1 resulted in lower wilting scores, indicating lower stress levels for the plants. In conclusion, the results emphasize the substantial influence of light intensity and spectral composition on both phenological and physiological traits of chickpea varieties. This underscores the critical importance of precise light management to enhance flowering and optimize yield outcomes.

### Multifactor and multivariable analysis on chickpea varieties under different light intensities

**Fig. 5:**
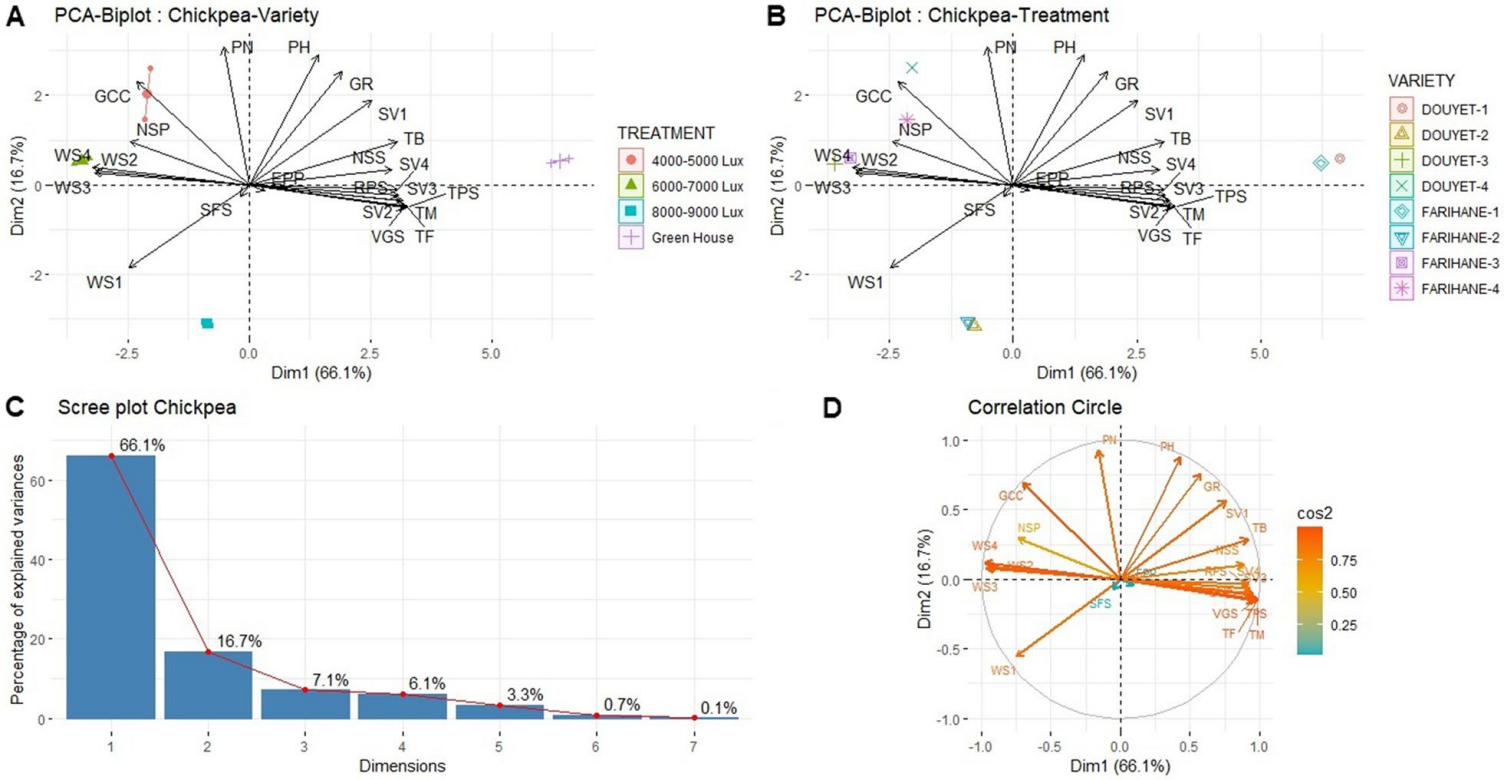
**A**–**D** Multivariable analysis on chickpea varieties under light intensity treatments. *PH* plant height, *TB* total biomass, *NSS* number of secondary stem, *PN* pods number, *EPP* empty pods percentage, *NSP* number of seeds per plant, *GR* growth rate, *GCC* green canopy cover, *SV1* seedling vigor 1, *SV2* seedling vigor 2, *SV3* seedling vigor 3, *SV4* seedling vigor 4, WS1 wilting score 1, wilting score 2, WS3 wilting score 3, *WS4* wilting score 4, *TF* time to flowering, *TPS* time of pod set, *TM* time to maturity, *VGS* vegetative stage length, *RPS* reproduction stage length, *SFS* seed filling stage length

A Principal Component Analysis (PCA) was conducted to analyze all variables for each light intensity treatment (T1: Green-house: 2000 µmol/m^2^/s; T2: 148–167 µmol/m^2^/s; T3: 111–129 µmol/m^2^/s; T4: 74–93 µmol/m^2^/s) in relation to the two chickpea varieties (Farihane and Douyet), considering all studied traits (Fig. 5). The PCA analysis revealed that PCA1 and PCA2 explained 66 and 17% of the total variation, respectively (Fig. 5c). The high cos2 values indicated good representation of variables near the circumference of the correlation circle, while low cos2 values suggested variables were not well represented by the main axes and were positioned closer to the center of the circle (Fig. 5d). Interestingly, the PCA plots demonstrated distinct correlation patterns for the studied traits depending on the light intensity and variety (Fig. 5b). Treatment 1 showed positive correlations with vegetative stage length, time of pods set, time to flowering, time to maturity, seed filling stage length, total biomass and number of secondary stems per plants, indicating a positive association with phenological stages and physiological traits but a negative association with stress-related traits. Conversely, to the treatment 2 and 3, which exhibited positive correlations with wilting scores 1, 2, 3, and 4, indicating a positive association with stress-related traits. On the other hand, treatments 4 displayed positive correlations with green canopy cover, pods number and number of seeds per plant, suggesting a positive relationship with plant growth and yield-related traits. In summary, the PCA analysis demonstrated that different light intensities had distinct effects on the correlations between phenological and physiological traits of chickpea varieties. Treatments 1, 2, 3, and 4 showed specific associations with different trait categories, highlighting the importance of managing light conditions effectively to optimize both phenological and physiological.

### Progression of seedling vigor and wilting score over time and between light intensity treatments

**Fig. 6:**
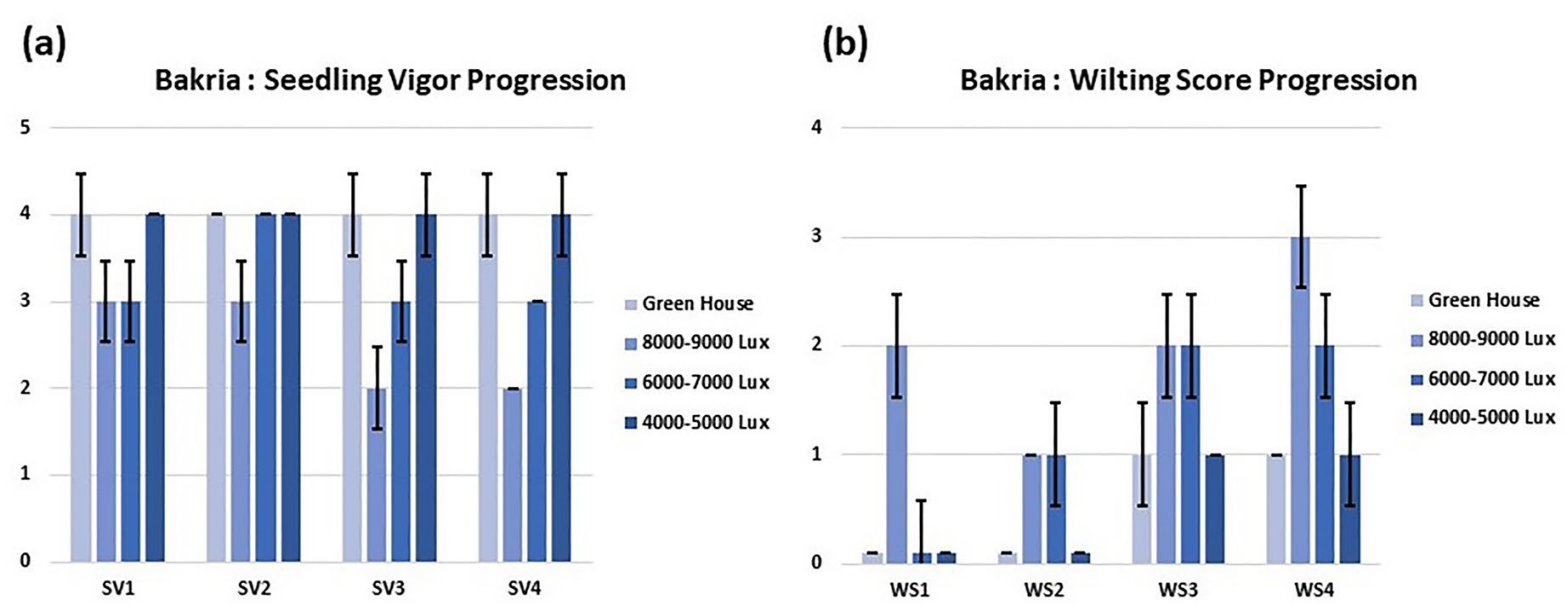
**(a)** Seedling vigor and (**b**) wilting score progression of Bakria variety over time. *SV1* seedling vigor at vegetative stage, *SV2* seedling vigor at flowering, *SV3* seedling vigor at reproduction stage, *SV4* seedling vigor at seed filling stage, *WS1* wilting score at vegetative stage, *WS2* wilting score at flowering, *WS3* wilting score at reproduction stage, *WS4* wilting score at seed filling stage

**Fig. 7:**
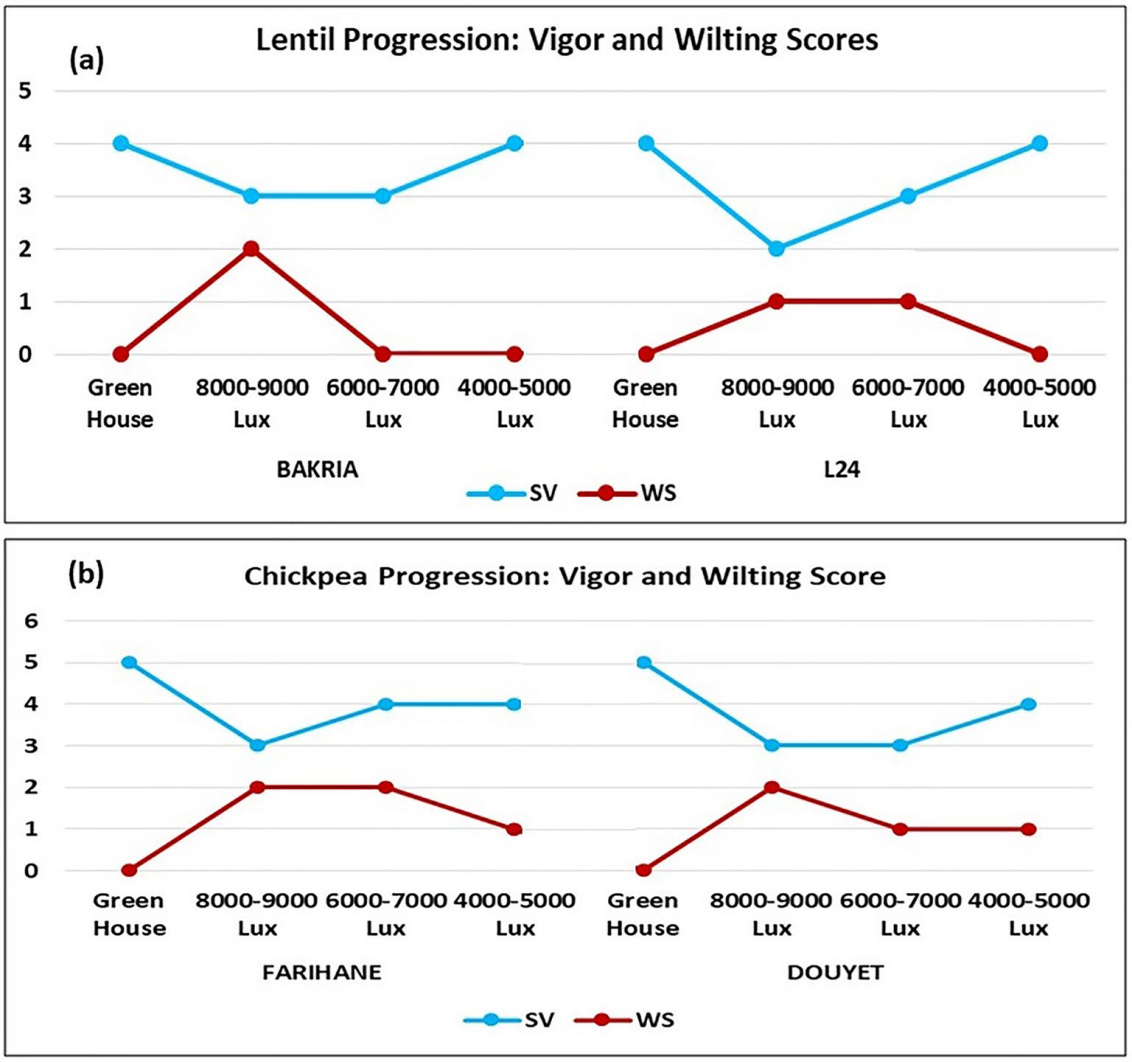
Seedling vigor and wilting score progression of (**a**) lentil and (**b**) chickpea in different light intensities. *SV* seedling vigor, *WS* wilting score

In this study, we investigated the progression of seedling vigor and wilting score over time, as well as the impact of different light intensity treatments on lentil and chickpea plants. Our results revealed significant trends in seedling vigor (Fig. 6a) and wilting score (Fig. 6b) across different development stages, from vegetative (SV1 & WS1) to flowering (SV2 & WS2), pod formation (SV3 & WS3), and maturity (SV4 & WS4) for lentil Bakria variety. Regarding seedling vigor, we observed a consistent decrease as the plants progressed from the vegetative stage to flowering, pod formation, and maturity. This suggests that the early stages of growth are characterized by higher vigor, which gradually declines as the plants reach maturity (Fig. 6). In contrast, the wilting score showed a consistent increase from the vegetative stage to flowering, pod formation, and maturity (Fig. 6). This indicates that the plants experienced increased susceptibility to wilting as they advanced in their growth stages, potentially due to increased light stress during these developmental phases. Furthermore, we examined the effects of varying light intensities on lentil and chickpea plants. Notably, for lentil plants, we found that an intensity of approximately 8000–9000 lux led to significantly reduced seedling vigor and elevated wilting scores compared to other light intensity treatments (Fig. 7). On the contrary, the other light intensity treatments showed higher seedling vigor and lower wilting scores. Except that, for the chickpea variety Farihane and lentil variety L24, the light intensity treatment of 6000–7000 lux exhibited similar results to the 8000–9000 lux treatment in terms of seedling vigor and wilting score (Fig. 7).

## Discussion

Photoperiod duration, light quality and quantity that a plant receives per day are important factors that determine suitable growth and development of plants [22, 23]. For speed breeding methods using extended photoperiod, the optimization of light parameters such as quality, intensity and photoperiod duration is very important, firstly to ensure a lower plant stress and mortality and secondly to accelerate the plant life cycles [16, 17]. Results of comparing the effect of different light intensity on lentil and chickpea growth and development was reported and discussed in this paper. Light intensity strongly influenced the expression of the majority of phenological and morpho-physiological traits, including the number of seeds per plant, growth rate, time to flowering, time of pod set, time to maturity, vegetative stage length, reproduction stage length, seed filling stage length, green canopy cover, total biomass, pods number, and plant height. While no significant differences on green canopy cover, the number of secondary stems, the percentage of empty pods and wilting score 1 for lentil, and on seed filling stage length and empty pods percentage for chickpea were observed.

**Fig. 8:**
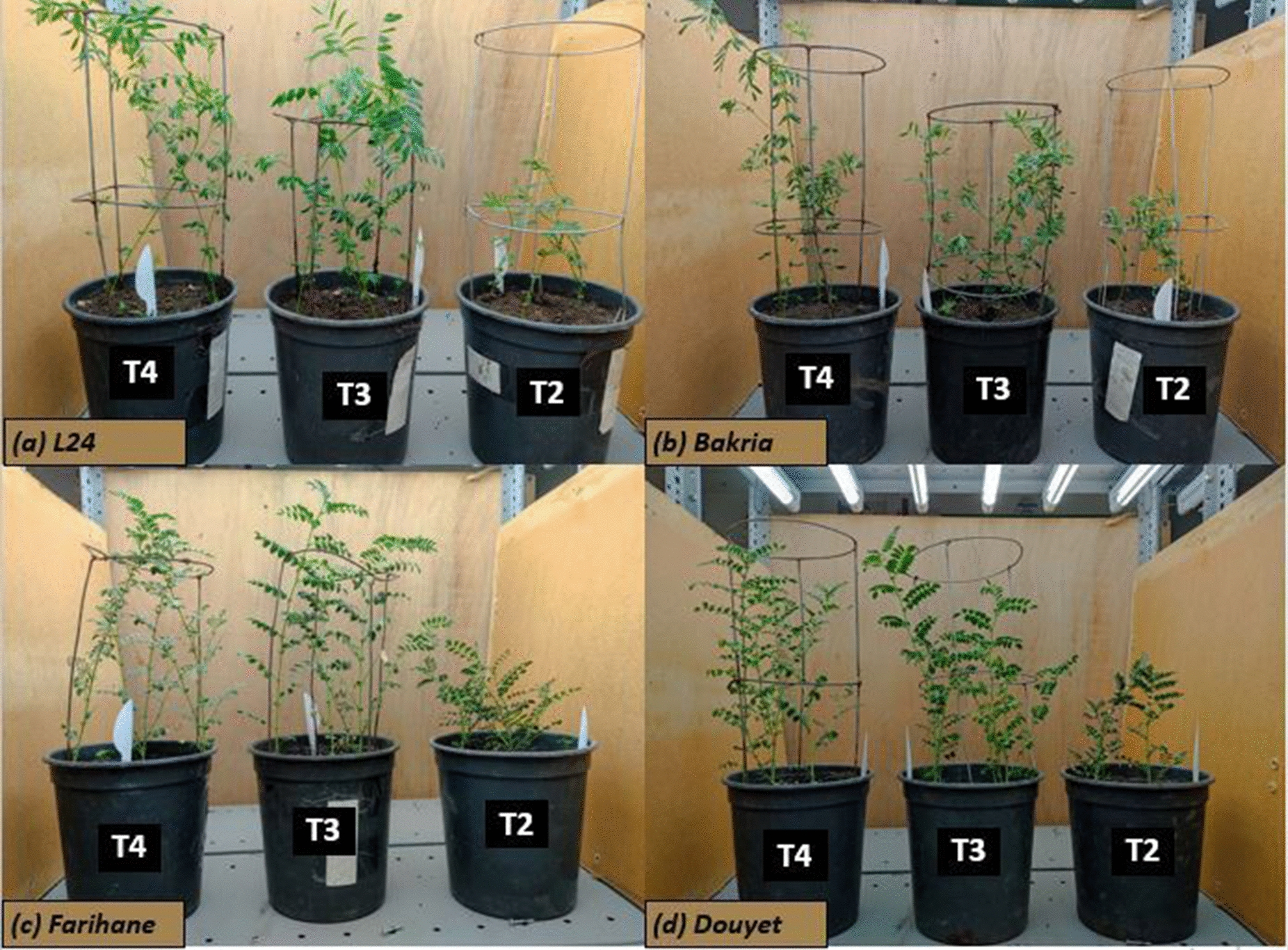
Light intensity treatments effect on lentil (**a**, **b**) and chickpea (**c**, **d**) varieties growth. T2 (treatment 2,148–167 µmol/m^2^/s), T3 (treatment 3, 111–129 µmol/m^2^/s), T4 (treatment 4, 74–93 µmol/m^2^/s)

Higher light intensity in treatment 2 (148–167 µmol/m^2^/s) influenced strongly and negatively the green canopy cover, plant height, seedling vigor, pods number, number of seeds per plant and plant height that illustrate (Fig. 8). This is because of the stress induced by the high intensity of light which produces many damage to photosynthesis reactions in the first degree [6], and can perturb the functioning of photosystems, reducing the efficiency of photosynthesis [24]. This disruption of photosynthetic reactions, in particular CO2 fixation, has an impact on plant morpho-physiological characteristics, while being positively related to stomatal limitations [25]. In contrast, treatment 4 (74–93 µmol/m^2^/s) enhanced clearly growth, phonological and yield-related traits in agreement with the results reported by [14] on Alfalfa (*Medicago sativa*) finding that lower intensity of light increased plant height and photosynthesis activity.

Various stress factors induce flowering in a large group of plant species. These plants exhibit flowering response as a consequent reaction to a set of distinct stress factors. Nevertheless, it is important to note that not all stressors induce flowering response in all plant species [26]. In our study, the flowering time has been significantly affected by treatment intensities comparing to green-house conditions (2000 µmol/m^2^/s) under natural day/night photoperiod without any light supplement with other treatments intensities under extended photoperiod conditions, for lentil and chickpea. The 2 treatments, 3 (111–129 µmol/m^2^/s) and 4 (74–93 µmol/m^2^/s) with medium level of intensity showed the earliest time to flowering (Tables 3 and 5) compared to treatment 2 that has a much higher intensity of (148–167 µmol/m^2^/s) that caused damage an d stress on plants. Thus medium and low level of light intensity were better to induce an early flowering than higher level of intensity, similar results were observed in *Perilla frutescens* by [27] reporting that flowering was 100% induced in 4 weeks under low light intensity. In contrast to these results, [28] have tested the effect of the light irradiation on flowering of Summer Pastels, and they found that a high level of intensity of 300 µmol/m^2^/s accelerated the flowering against low level of intensity of 100 µmol/m^2^/s. According to [29], the enhanced growth is attributed to a combination of improved light utilization efficiency under low light conditions and extended daylight duration.

In summary, this study has provided significant information on the impact of light intensity on morphological, phonological and physiological traits in lentil and chickpea. Despite the limited availability of comparable studies for specific traits and the studied factors, our results highlight the crucial importance of taking into account the complex interactions between plant traits and light intensity especially for speed breeding purposes. The absence of a solid reference base for certain trait categories further highlights the ongoing need for targeted research to better understanding these aspects. The results generated in this study will serve as a valuable starting point for future research into the underlying mechanisms of plant responses to light intensity, especially for lentil and chickpea. Ultimately, the results presented here make a significant contribution to the optimization of a speed breeding method allowing higher genetic gain thanks to shorter plant growth cycle with limited stress and mortality. In fact, the major implication of limited stress and mortality under the optimized light intensity is that it could help to obtain F_6_ populations with higher genetic diversity as result of higher population size, and to achieve rapidly higher homozygosity in segregating populations obtained from crosses. This would contribute to develop training populations for genetic studies (recombinant inbred lines for instance) and feed the breeding pipelines with new lines that were fixed rapidly using limited resources in the perspective of developing new varieties.

## Conclusion

This experience provides useful information for optimizing the speed breeding protocol for food legumes such as lentil and chickpea. It is clear that duration of plants light exposure (extended photoperiod) affects significantly, the plants growth and development [16]. In this study, the light intensity also has influenced the plant growth and development, and this is clearly showed in the results. The high intensity (148–167 µmol/m^2^/s) was the most stressful and this was shown by dwarf plants, high wilting severity, low seedling vigor, late flowering, high percentage of empty pods, low pod number and low green canopy cover. While modest intensities in treatment 3 (111–129 µmol/m^2^/s) and treatment 4 (74–93 µmol/m^2^/s) has showed contrasted results. The application of adequate light intensity combined with optimized light duration and light quality, would therefore ensure rapid generation turnover for these crops, with limited loss of genetic variability.

## Data Availability

The datasets used and/or analyzed during the current study are available from the corresponding author on reasonable request.
